# An Alternative Approach to Sex Offenders

**Published:** 1989-08

**Authors:** Hugh W Gilbert, J Clive Gingell

**Affiliations:** Andrology Research Unit, Department of Urology, Southmead Hospital, Westbury-on-Trym, Bristol BS10 5NB; Andrology Research Unit, Department of Urology, Southmead Hospital, Westbury-on-Trym, Bristol BS10 5NB


### An Alternative Approach to Sexual Offenders

**Hugh W Gilbert FRCS**
**J Clive Gingell FRCS**
Andrology Research Unit, Department of Urology, Southmead Hospital, Westbury-on-Trym, Bristol BS10 5NB

### INTRODUCTION

That which constitutes a sexual offence is defined by the norms of society. Although these norms may change in some minor respects and although sexuality is surrounded by ignorance, guilt, inhibition and taboo, transgression is invariably clear. Men who commit sexual offences are very likely to reoffend whether they are imprisoned or not, indeed, the minor offender may progress to more serious crimes. Newspapers frequently remind us that current treatment, where used, is failing to prevent and protect against the recidivist. Pharmacological inhibition of sexuality has been investigated for many years. Difficulties with side-effects, compliance and ethical objections have prevented a comprehensive investigation of this so-called chemical castration. Our experience with the use of a Luteinising Hormone Releasing Hormone (LHRH) analogue in the treatment of carcinoma of the prostate has led to an interest in it's possible role in the treatment of sexual offenders.

### THE PROBLEM

In 1987 the police recorded 25,200 notifiable sexual offences. This figure includes cases of indecent assault, exposure, rape, homosexual and heterosexual paedophilia and incest (1). The subsequent trend, unlike that for all other crimes except violence against the person, is upward. 6,200 men were sentenced for indictable offences; 36% were taken into custody. In 1984, 28% of those men who were sentenced to imprisonment for sexual offences were reconvicted within two years (2). 52% of all convicted sexual offenders have had at least one previous conviction (3). A custodial sentence, therefore, is neither treating nor effectively deterring sexual offenders. In prison, these men are isolated from other offenders because of the risk of physical injury. By associating with one another their fantasies may be reinforced. Apart from removing the offender from society the main benefit of custodial care lies in the opportunity provided for close supervision and monitoring of the response of the offender to treatment.

### MEDICAL CASTRATION

The role of testosterone in relation to sexuality is clear in animals. The higher the level of androgen then the higher the sexual activity and, furthermore, the level of aggression increases. This relationship is not as well defined in humans. Although the mean level of testosterone has been shown to be higher in violent sexual offenders, the values all fall within the normal range (4). The first attempts to alter sexual drive by means of drugs occured more than 40 years ago. Oestrogen preparations were used but the side-effects of this treatment, including severe nausea, irreversible gynaecomastia, testicular atrophy, the risks of osteoporosis and thrombo-embolic disease, could not be justified (5). Subsequently interest focused on the use of psychotropic drugs. In 1974 a double blind trial of two of these preparations was conducted and showed that no clinically useful reduction in sexual behaviour was obtained (6). Cyproterone Acetate (CPA) was first synthesised in the early 1960s and, once it's powerful anti-androgenic properties were recognised, it's use in the treatment of sexual deviation was investigated. A daily dose of 100–300 mg leads to the eradication of sexual drive. The clinical actions of CPA, when used in this role, were reviewed by Kockott (7). Plasma levels of testosterone are reduced to castrate levels. Whilst sexual drive can be completely removed the ability to achieve erection may not necessarily be totally suppressed. Sexual fantasies only diminish over a period of several months treatment. Some effects, such as altered affect or lack of concentration may occur in the first few weeks of treatment but these are transient. The loss of sexual drive, erectile ability, ejaculation and spermatogenesis are completely reversible on cessation of therapy. Gynaecomastia occurs in only approximately 15% of patients treated.

It would therefore seem, with certain reservations, that CPA has a role in the reduction of sexual drive. If this were true then it might be used as adjunctive therapy in the overall management of sexual offenders. In the only controlled trial to date, a significant reduction in the frequency of sexual ideas and activity was reported in a group of sexual offenders treated with CPA (8). In addition it was found to reduce the sexual response to erotic stimulation. The reaction to erotic films was, however, unaffected. Despite a series of promising trials in the early 1970s this treatment fell out of favour. The principle reason for this, however, would seem to us to be more ethical than pharmacological.

The discovery of the structure of Luteinising Hormone Releasing Hormone (LHRH) allowed the manufacture of many powerful analogues. Initially their intended role was to be in the treatment of female infertility, however, clinical trials showed that these compounds paradoxically produce inhibition of normal gonadal function when administered continuously. The mechanism is complex (9). Investigation of the possible use of LHRH analogues as male contraceptives have shown that although spermatogenesis is abolished it is at the expense of sexual potency and drive. Despite this there was no evidence of systemic toxicity and, indeed, all men were able to continue with normal physical activities including sports (10). LHRH analogues have the advantage that they can be given as monthly depot injections. All effects are reversible following cessation of treatment. There is no recorded experience of it's use in the management of sexual offenders.

The current treatment of advanced carcinoma of the prostate requires the elimination of androgen activity. This can either be achieved by surgical castration or by pharmacological methods. At this hospital all men with metastatic carcinoma of the prostate are treated either with CPA, an LHRH analogue, or a combination of both with the desired effect of reducing the serum testosterone to the castrate range. This treatment is complicated by the onset of sexual impotence and the loss of libido. Of 34 men who were potent prior to treatment all were rendered impotent by their therapeutic castration. We have shown in this group of patients that erectile function can be restored with the use of intracavernosal papaverine injection (ICP) (11). Despite this only two men wanted ICP, the remainder having lost all sexual interest. The two men who continued to use ICP did so, not because their libido was preserved, but because they were motivated by considerably younger partners. Thus, albeit in an older age group, the reduction of testoterone produces a reduction in sexual drive.

### DISCUSSION

We believe that this effect should be investigated in the treatment of sexual offenders. Informed consent should not be the subject of any ethical reservations. If treatment that includes pharmacological castration is made a condition of release from prison this surely avoids the dilemma. The indications for this type of therapy have previously been the subject of considerable controversy and close co-operation and collaboration is required between psychiatrists, psychologists and the legal profession in order to establish the guidelines that are so obviously needed. Whereas the harm caused by, and hence the need to treat, rape or paedophilia is clear; that caused by sexual eccentricities may be less so. The offenders insight into his disease may affect the method of psychological therapy but is immaterial to the effect of anti-androgenic treatment. Imprisonment of the sexual offender without any form of therapy reinforces their sexual fantasies so that when they are released not only are they likely to reoffend but frequently do so in a far more serious manner. Any therapeutic option that may prevent this and hence protect not only the offender but also his victims must be worthy of urgent examination.

If this treatment is to be further investigated an effective method of monitoring the response must be developed. To date the mainstay of evaluation has been psychological assessment and reassessment both by direct questioning and by questionnaire. In recent years there has been a revolution in the understanding of the physiology of penile erection. This is as a result of the development of more sophisticated investigative techniques. Detailed information not only on penile tumescence but also on erection quality including rigidity is now obtainable. This allows the response to erotic stimuli to be recorded in an accurate and reproducible manner. In addition, with ambulatory equipment the effect of everyday events can be assessed. Nocturnal penile rigidity is believed to reflect erectile activity without psychological or voluntary inhibition and again is easily measured (13). A combined approach of both psychological and physical assessment is therefore envisaged.

### CONCLUSION

The current method of managing sexual offenders by imprisonment is dangerously inneffective. The release back into society of untreated, unsupervised sexual offenders is not acceptable. In order to protect society from a high rate of recurrent offenders advantage should be taken of investigating newer treatment modalities. Surgical castration, although effective, will never gain favour on ethical grounds. Oestrogen therapy is feminising and causes cardio-vascular complications. The recently available LHRH analogues are effective in reducing sexual drive, can be given by depot injection and therefore there are no difficulties with patient compliance. There are no troublesome side-effects. The use of these drugs is seen as an adjunct to psychological treatment and should be conducted in appropriate custodial care. Treatment would continue until it was proven by psychological and physical means to be effective.

Unless effective treatment is offered to sexual offenders members of the public will continue to remain vulnerable. Medical conviction and perhaps of more importance, the political will is required to help control this increasing social problem.

### REFERENCES

1. Criminal Statistics: England and Wales (1987) Command Paper 498. London: HMSO, 1988.
2. Prison Statistics: England and Wales (1987) Command Paper 547. London: HMSO, 1988.
3. CRAFT, M. (1981) Sex Offences: The size of the problem and treatment. *Update* **23**, 1549–1557.
4. RADA, R. T., LAWS, D. R. and KEZINER, R. (1976) Plasma testosterone levels in the rapist. *Psychosom Med.* **38**, 257–258.
5. GOLLA, F. L. and HODGE, S. R. (1949) Hormone treatment of sex offenders. *Lancet* **1**, 1006.
6. TENNENT, G., BANCROFT, J. and CASS, J. (1973) The control of deviant sexual behaviour by drugs: a double blind controlled study of benperidol, chlorpromazine and placebo. *Arch. Sex Behav.* **3**, 261–272.
7. KOCKOTT, G. (1983) The treatment of sexual delinquency with antiandrogens. *Psychiatr. Prax.* **10**, 158–164.
8. BANCROFT, J., TENNENT, G., LOUCAS, K. and CASS, J. (1974) The control of deviant sexual behaviour by drugs. *Br. J. Psychiatry* **125**, 310–315.
9. SANDOW, J. (1983) Clinical applications of LHRH and its analogues *Clin. Endocrinol* **18**, 571–592.
10. SHURMEYER, T. H., KNUTH, U. A., FREISCHEM, C. W., SANDOW, J., BINT AKHTAR, F. and NIESHLAG, E. (1984) Suppression of pituitary and testicular function in normal men by constant Gonadotrophin-Releasing Hormone agonist infusion. *J. Clin. Endocrinol Met.* **59**, 19–24.
11. GILBERT, H. W., GILLATT, D. A., DESAI, K. and GINGELL, J. C. (1988) Intracorporeal papaverine injection in androgen deprived men. Proceedings of the Third Biennial World Meeting on Impotence. Abstract 178.
12. ALLEN, R. P. (1981) Erectile impotence: Objective diagnosis from sleep related erections (nocturnal penile tumescence). *J. Urol.* **126** 353.

